# Identification of suitable sites for mountain ginseng cultivation using GIS and geo-temperature

**DOI:** 10.1186/s40064-016-2031-x

**Published:** 2016-03-31

**Authors:** Hag Mo Kang, Soo Im Choi, Hyun Kim

**Affiliations:** Department of Forest Environmental Science, Chonbuk National University, Jeonju, 54896 South Korea; Department of Forest Resources, Sunchon National University, Suncheon, 57922 South Korea; Jeollabuk-do Forest Environment Research Institute, Jinan, 55454 South Korea

**Keywords:** Automatic weather station (AWS), Cultivated mountain ginseng (CMG), Geographic information system (GIS), *Panax ginseng*

## Abstract

This study was conducted to explore an accurate site identification technique using a geographic information system (GIS) and geo-temperature (gT) for locating suitable sites for growing cultivated mountain ginseng (CMG; *Panax ginseng*), which is highly sensitive to the environmental conditions in which it grows. The study site was Jinan-gun, South Korea. The spatial resolution for geographic data was set at 10 m × 10 m, and the temperatures for various climatic factors influencing CMG growth were calculated by averaging the 3-year temperatures obtained from the automatic weather stations of the Korea Meteorological Administration. Identification of suitable sites for CMG cultivation was undertaken using both a conventional method and a new method, in which the gT was added as one of the most important factors for crop cultivation. The results yielded by the 2 methods were then compared. When the gT was added as an additional factor (new method), the proportion of suitable sites identified decreased by 0.4 % compared with the conventional method. However, the proportion matching real CMG cultivation sites increased by 3.5 %. Moreover, only 68.2 % corresponded with suitable sites identified using the conventional factors; i.e., 31.8 % were newly detected suitable sites. The accuracy of GIS-based identification of suitable CMG cultivation sites improved by applying the temperature factor (i.e., gT) in addition to the conventionally used factors.

## Background

The South Korean society in the twenty-first century is characterized by a high standard of living and diversified leisure activities, driven by continuous economic growth and progress in cutting-edge technologies. In line with the changing trends of lifestyle and leisure, there is growing interest in health, and eco-food from unpolluted areas and medicinal herb resources are increasing in popularity and demand. In particular, unpolluted forest products from South Korean mountains have taken center stage, because of suspicion and mistrust regarding heavy-metal contamination of imported forest products and medicinal herbs.

In South Korea, forests extend over 6,369,000 ha (at end 2010) across the country and occupy 63.7 % of the entire territory, the fourth highest forest-to-land ratio among the OECD countries after Finland (72.9 %), Sweden (68.7 %), and Japan (68.5 %). When classified according to ownership, private forests have the largest portion (68.1 %; 4,338,000 ha), followed by national forests (24.2 %; 1,543,000 ha), and public forests (7.7 %; 488,000 ha). As of the end of 2013, the number of forest land owners was 2,379,000 (1,046,000 resident; 1,333,000 non-resident). The average forest area per forest land owner was as small as 1.8 ha, with owners of <0.5 ha accounting for 56 % (Korea Forest Service 2014d). Because of this small-scale ownership pattern, South Korea’s forest management faces difficulties with systemization despite its fourth highest forest-to-land ratio among the OECD countries. As if to reflect this reality, from the annual gross forest product in 2013 (6612 million USD), the proportion of timber products was as low as 5.6 % (373 million USD), with the revenues of forest land owners heavily relying on non-timber products, such as landscape materials (11.1 %; 731 million USD), orchard products (10.4 %; 687 million USD), medicinal herbs (7.1 %; 473 million USD), wild edible greens (5.4 %; 355 million USD), and mushrooms (12.9 %; 188 million USD). The cultivation amounts of and revenues from non-timber forest products have been constantly increasing over recent years.

Among the medicinal herbs cultivated in South Korea, ginseng (*Panax ginseng* Meyer) has been reported in Chinese and South Korean classics of Eastern medicine as the most effective energy booster, and is primarily used as a herbal remedy to cure energy depletion. For ages, ginseng has been used for enhancing physical fitness and recovering from fatigue, as well as controlling digestive, nervous, metabolic, and circulatory functions (Nam 2002). In South Korea, systematic ginseng cultivation in natural mountainous environments began about 10 years ago. As of 2013, the total cultivation area amounted to 8722 ha and the number of cultivators was 2449. The annual products and revenues amounted to 26 tons and 30,856 thousand USD (1186.77 USD/kg), with the cultivation area growing rapidly each year (Korea Forest Service 2014c).

The Korea Forest Service (KFS) designated cultivated mountain ginseng (CMG) as a special forest product and controls its cultivation and management. According to the KFS definition, CMG is “ginseng products (including dried products) that are cultivated in forest land without the use of man-made structures and facilities such as sun shades as per the Forest Management Act Article 2 Paragraph 1.” Additionally, since 2011, CMG cultivation in South Korea is subject to legal requirements such as production registration with the pertinent agency and production feasibility tests, as well as pre-sale quality inspection (Korea Forest Service 2011). With the superior efficacy of CMG gaining a worldwide reputation, CMG export has drastically increased from 1.1 ton (69,100 USD) in 2012 to 94.5 ton (19,807,900 USD) in 2014 (Korea Forest Service 2014a). In 2014, the main importing countries were China (39.4 ton), Taiwan (29.4 ton), Hong Kong (15.4 ton), Japan (5.0 ton), and the United States (2.4 ton) (Korea Forest Service 2014b). In order to boost the income of a CMG cultivation farm household, continuous production of high-quality CMG should be ensured. First, a suitable site should be identified, which is a challenge given the extremely high sensitivity of CMG plants to the environmental conditions in which they grow (Woo and Lee 2002; Seo et al. 2007). This problem can be effectively addressed by using a geographic information system (GIS) (Beon et al. 2013).

Studies have been conducted to identify suitable sites for the cultivation of red pepper (*Capsicum annuum*) (Jung et al. 2004), black raspberry (*Rubus coreanus*) (Kim and Lee 2006; Lee et al. 2007), Omija (*Schisandra chinensis*) (Kim et al. 2011a), and mulberry (*Morus alba*) (Kim et al. 2012a) by using GIS and analyzing the suitability of the localities taking into account factors such as the soil and topographic features. One study explored a method for identifying suitable sites for ginseng cultivation by using soil, geographic, climatic, hydrologic, and topographic characteristics as factors (Kim 2002); however, detailed cultivation-related features of each factor could not be determined. Studies on identifying suitable sites for CMG cultivation have also been conducted using soil, topography, and light as factors (Beon et al. 2013; Han 2014). Interestingly, only a few studies have examined temperature conditions, one of the most important factors for crop cultivation. Some studies have dealt with temperature for apple, garlic, winter wheat, and tea crops (Kwon et al. 2004; Kim et al. 2009, 2011b, 2012b; Wang et al. 2011; Li et al. 2012), but not for identifying suitable sites for CMG cultivation.

This study was conducted to identify suitable sites for CMG cultivation using both a previously described method and a newly developed method, in which temperature, one of the most important factors for crop farming, was added an additional factor. We also compared the results of the 2 methods to develop an accurate method for identifying CMG cultivation sites.

## Methods

### The study site

**Fig. 1:**
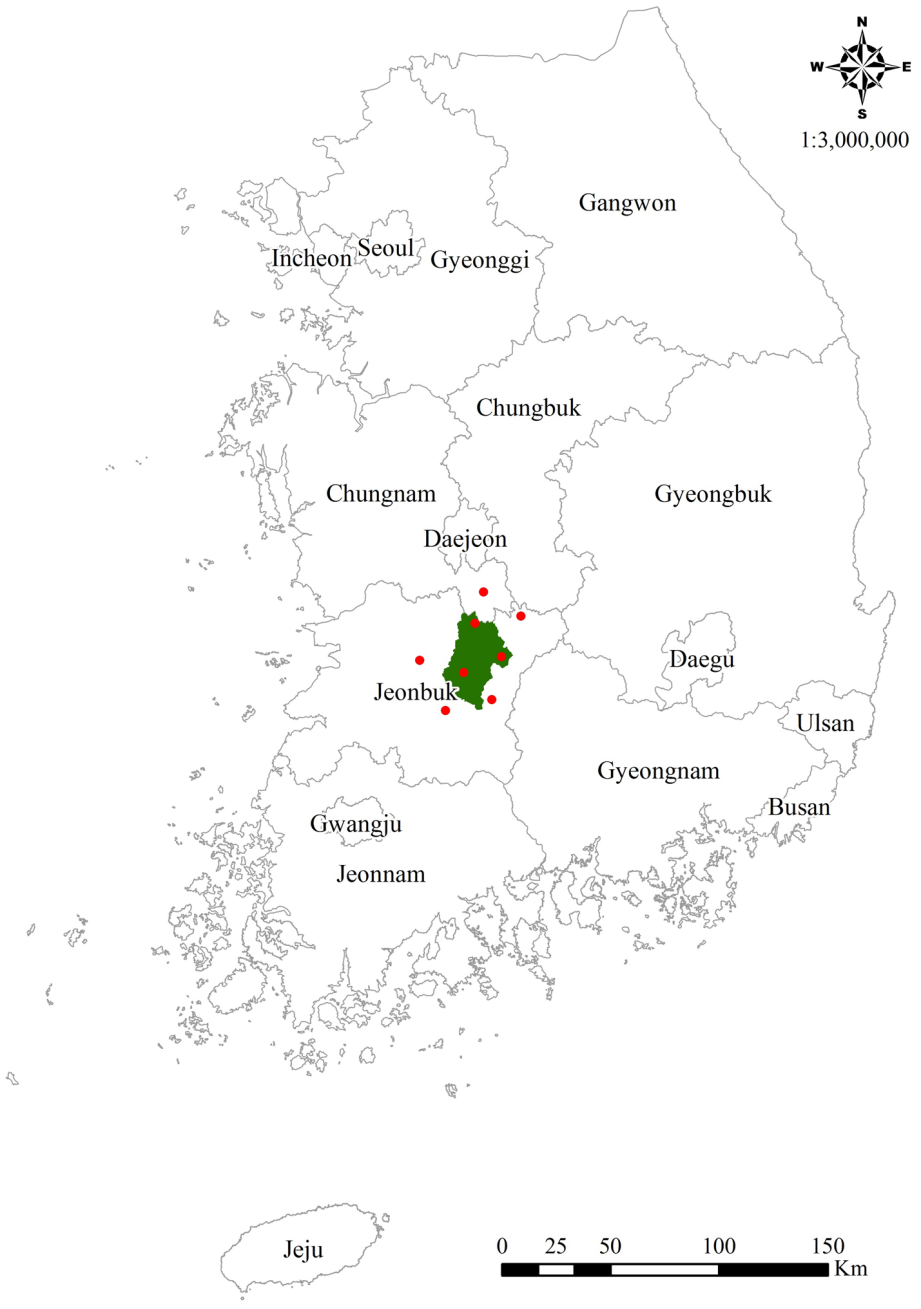
The location map of the study site. *Green color* indicates the targeted study site (Jinan-gun). This site is located in the eastern mountainous region of Jeollabuk-do (Jeonbuk), South Korea. The locations of the automatic weather stations (AWSs) used in this study are marked as *red points*

The study site, Jinan-gun, is located in the eastern mountainous region (elevation: 600–1100 m) of Jeollabuk-do, South Korea (Fig. 1). According to the land register of Jinan-gun as of December 31, 2013, cultivated land (rice paddies, crop fields, and orchards) accounts for only 11.2 % of the total area (8839.7/78,912.0 ha), whereas the proportion of forest land is 77.4 % (61,110.0/78,912.0 ha) (Jinan-gun 2015). In particular, according to the internal data of the Jeollabuk-do Provincial Government regarding the CMG production declaration between July 2011 and July 2013, the total area of CMG cultivation sites in Jinan-gun was 531.6 ha, accounting for 6.1 % of all CMG cultivation sites in South Korea (8722.0 ha) and 47.4 % of the total CMG cultivation area of Jeollabuk-do (1121.3 ha; 246 farm households).

### Factors for identifying suitable sites

We identified suitable sites for CMG cultivation using the factors including conditions described in a previous study (Beon et al. 2013), namely elevation, aspect, slope, forest type (tree species), organic content, effective soil depth, soil humidity, and drainage. Suitable sites were also identified using temperature as an additional factor influencing CMG growth. The results of the 2 methods were subsequently compared and evaluated.

### Data processing

The GIS-based data for the detection of suitable CMG cultivation sites were processed as grid-shaped raster data. First, the geographic data required [elevation, also known as Digital Elevation Model (DEM), aspect, and slope] were generated using numerical maps (1:25,000; National Geographic Information Institute). The 2010 5th Forest Type Map (1:25,000; Korea Forest Service) was used to derive data for forest type (tree species), which was used to distinguish bamboo forest and un-stocked forest land from the stocked forest land, and the Forest Soil Digital Map (1:25,000; Korea Forest Service) was used to generate data on organic content, effective soil depth, soil humidity, and drainage. Finally, the raster data for identifying suitable CMG cultivation sites were reclassified after applying weights according to the conditions of each factor. The spatial resolution used in the study (10 m × 10 m) was higher than that of a previous study (30 m × 30 m) (Beon et al. 2013). This spatial resolution of 10 m × 10 m refers to the area of each grid cell of the suitable cultivation site, which corresponds to an actual CMG cultivation area of 100 m^2^.

### Generation of geo-temperature

Geo-temperature (gT; °C) indicates the elevation-dependent temperature change, taking into account the temperature lapse rate (TLR; °C/m) with respect to elevation (EL; m), base elevation (BaEL; m), and base temperature (BaT; °C) (Eq. 1). The TLR generally applied in South Korea is 0.6 °C per 100 m (0.6/100 °C/m; Ann et al. 2007). When EL is higher than BaEL, gT decreases from BaT by the difference between EL and BaEL times TLR. However, when EL is lower than BaEL, gT increases from BaT by the difference between EL and BaEL times TLR.

1 $$gT = BaT - \left( {EL - BaEL} \right) \times TLR$$

The gT was generated using temperature data collected from the automatic weather stations (AWSs) of the Korea Meteorological Administration. The AWSs from which the data were collected are installed in 3 locations in Jinan-gun (Jinan-eup, Jucheon-myeon, and Donghyang-myeon) and 5 locations in areas surrounding Jinan-gun (Jeonju-si, Geumsan-gun, Imsil-gun, Jangsu-gun, and Muju-gun) (Fig. 1). The temperatures measured at the 8 AWSs were averaged over 3 years (2012–2014) to derive a temperature range matching the gT for suitable sites for mountain ginseng cultivation. Temperatures at each AWS elevation were converted into reference temperatures (elevation = 0 m) using the equation for the gT. The reference temperature was entered as attribute factors after generating the 8 point data using the AWS coordinates provided by the Korea Meteorological Administration. The 8 point data underwent spatial interpolation, and the elevation-dependent gT was calculated using the DEM. For spatial interpolation, ordinary kriging of spherical and semi-variogram functions was used (Kim et al. 2011b, 2012b).

### Factors, conditions, and weights for identifying suitable cultivation sites

Factor combination and linear combination techniques are often used to determine suitable cultivation sites in combination with GIS (Jo et al. 2001; Kim and Lee 2006), and the two techniques can be differentiated by how each method assigns the weighted values to the factors. The factor combination technique assigns the weighted value of 1 to the best condition for each factor used to determine a suitable cultivation site and assigns the weighted value of 0 for the remaining conditions. Meanwhile, in the linear combination technique, the weighted value of 1 is assigned to the best condition for each factor, and differentiated weighted values are assigned to the next best conditions for each factor based on the following Eq. 2.

2 $$Weight_{k} = \frac{1}{n} \times \left( {n - \mathop \sum \limits_{k = 1}^{n} \left( {k - 1} \right)} \right)$$

Weight_k_ indicates the weighted value for the condition of the kth rank within the factor; n indicates the number of conditions within the search factor for a suitable cultivation site; k indicates the rank of the condition.

CMG cultivation can be divided into the following stages: preparation (searching for the candidate location, cultivation feasibility test), cultivation site construction (canopy trimming, removal of shrubs and undergrowth, tilling), seed management (pulp removal, germination, germinated seed management), sowing and planting (mature fruit seeding, germinated seed planting, seedling transplantation), cultivation management (prevention of damage from theft and wild animals, lower vegetation management), harvest, and storage. This study deals with the first stage of searching for a cultivation site; specifically, selection of major factors to be considered in identifying new suitable sites. In this study, important factors for identifying suitable CMG cultivation sites were annual average and summer temperatures, as well as temperatures during germination, sprouting, leaving, and flowering/fruiting (Jeon et al. 2013), in addition to the 9 factors used in a previous study (Beon et al. 2013). Moreover, conditions for each selected factor were categorized as possible variants, and weights were assigned to the specific factor categories for use in identifying cultivation sites (Table 1).

**Table 1:** Factors, conditions, and weights for the detection of suitable cultivation sites

Factor	Condition	Weight
Elevation	More than 300 m	1
	Others	0.5
Aspect	North	1
	East, Northeast, Northwest	0.67
	Others	0.33
Slope	<30°	1
	Others	0.5
Forest type (tree species)	Hardwood forest*, Larix kaempferi*	1
	Mixed forest	0.67
	Others	0.33
Soil organic content	2–9 %	1
	Others	0.5
Effective soil depth	More than 15 cm	1
	Others	0.5
Soil texture	Sandy loam, loam, silty clay loam	1
	Others	0.5
Soil humidity	Suitable humidity	1
	Other	0.5
Drainage	Good condition	1
	Others	0.5
Annual average temperature (Jan 1–Dec 31)	0–10 °C	1
	Others	0.5
Summer temperature (Jul 1–Aug 31)	20–25 °C	1
	Others	0.5
Temperature during germination (Mar 1–Mar 20)	5–15 °C	1
	Others	0.5
Temperature during sprouting (Mar 21–Apr 10)	5–15 °C	1
	Others	0.5
Temperature during leafing (Apr 11–May 10)	10–20 °C	1
	Others	0.5
Temperature during flowering/fruiting (May 11–Jun 30)	20–25 °C	1
	Others	0.5

### Detection of suitable cultivation sites and comparative evaluation

Suitable cultivation sites were identified using the linear combination technique as in a previous study (Beon et al. 2013). Beon et al. (2013) demonstrated that the linear combination technique resulted in a higher accuracy of the determination of suitable cultivation sites than the factor combination technique. Identification using the previous method applying the conventional factors and the new method including temperature as an additional factor was performed. For a comparison of two methods, to reclassify the analyzed data using the linear combination technique, an equal-interval data classification method was used to reclassify the detection performance into 4 grades: suitable site, possibly suitable site, probably unsuitable site, and unsuitable site. The cultivation sites are typically reclassified into 3 (Kim and Lee 2006; Lee et al. 2007) or 4 (Jung et al. 2004; Kim et al 2011a, 2012a; Li et al. 2012), or rarely, 5 (Wang et al. 2011) grades. This study categorized the analyzed cultivation sites into 4 grades, which is a commonly used reclassification method and consistent with the previous study that searched for suitable cultivation sites of CMG (Beon et al. 2013). Meanwhile, an equal-interval data classification method was used as a reclassification method, emphasizing the amount of an attribute value relative to other values (ESRI 2012).

The sites identified as 4 grades by each of the 2 methods were separately extracted and compared by overlaying. Additionally, the comparative analysis was performed by overlapping the results of the 2 methods, as well as overlapping their respective identified areas and actual CMG cultivation sites (*n* = 53). As main analysis software, ArcMap 10.1 (ESRI Inc., Redlands, CA, USA) was used, and the area comparison and evaluation was made using the Zonal Statistics Tool of the Spatial Analysis Tools.

## Results and discussion

### Geo-temperature

**Fig. 2:**
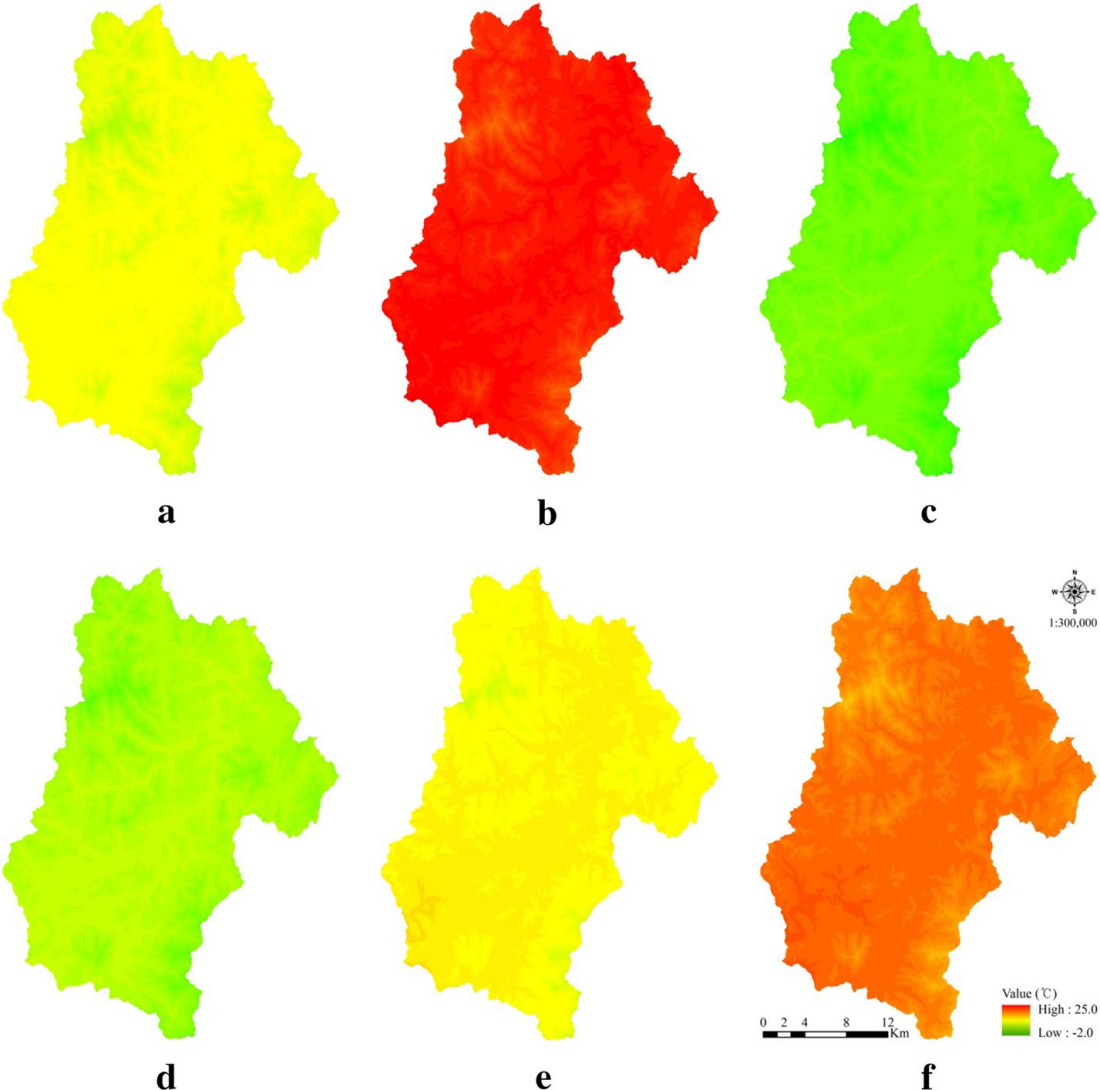
The geo-temperature maps for each temperature factor. **a** Annual average temperature, **b** summer temperature, **c** germination stage temperature, **d** sprout stage temperature, **e** leafing stage temperature, **f** flowering/fruiting stage temperature

Table 2 presents the 3-year average temperatures for each factor at the 8 AWSs, elevation of each AWS, and reference temperatures converted with respect to the elevation of 0 m. The highest average temperatures and elevation-dependent reference temperatures occurred at Jeonju-si, followed by Geumsan-gun and Muju-gun (Table 2). The gT was generated for each factor of the entire Jinan-gun site via spatial interpolation using the reference temperature of each factor. The gT ranges for Jinan-gun were 5.6–11.5, 18.9–24.9, −1.1 to 4.4, 2.1–7.8, 7.4–13.2, and 14.4–20.3 °C for annual average temperature, summer temperature, germination, sprouting, leaving, and flowering/fruiting stage temperatures, respectively. The study site was a mountainous region with an elevation of 600–1100 m, and the geo-temperature maps clearly reflected the relative change of gT according to change in elevation: gT decreased at higher elevations, and increased at lower elevations (Fig. 2).

**Table 2:** Reference temperature for each temperature factor (average temperature)

Temperature factor	Temperature for each AWS location (elevation) (°C)							
	Jinan-eup (351.4 m)	Jucheon-myeon (259.0 m)	Donghyang-myeon (320.2 m)	Jeonju-si (53.4 m)	Geumsan-gun (170.4 m)	Imsil-gun (247.9 m)	Jangsu-gun (406.5 m)	Muju-gun (205.8 m)
A	12.8 (10.7)^a^	12.0 (10.4)	12.1 (10.2)	14.0 (13.6)	12.7 (11.7)	12.6 (11.1)	13.1 (10.6)	12.5 (11.3)
B	26.2 (24.1)	25.3 (23.8)	25.7 (23.7)	27.1 (26.8)	26.4 (25.4)	25.9 (24.5)	26.2 (23.8)	26.2 (24.9)
C	5.9 (3.8)	5.5 (3.9)	5.3 (3.4)	6.5 (6.1)	5.6 (4.6)	5.3 (3.8)	6.4 (3.9)	5.7 (4.5)
D	9.4 (7.2)	8.5 (7.0)	8.5 (6.6)	9.8 (9.5)	9.2 (8.2)	8.6 (7.1)	9.5 (7.0)	9.1 (7.8)
E	14.6 (12.4)	13.9 (12.3)	14.0 (12.0)	15.3 (15.0)	14.6 (13.5)	14.0 (12.5)	15.1 (12.6)	14.5 (13.3)
F	21.7 (19.6)	20.9 (19.3)	21.1 (19.2)	22.6 (22.3)	21.9 (20.9)	21.4 (19.9)	22.0 (19.6)	21.5 (20.3)

A: Annual average temperature (Jan 1–Dec 31), B: Summer temperature (Jul 1–Aug 31), C: Temperature during germination (Mar 1–Mar 20), D: Temperature during sprouting (Mar 21–Apr 10), E: Temperature during leafing (Apr 11–May 10), F: Temperature during flowering/fruiting (May 11–Jun 30)

^a^Figures are base temperatures (°C), and the figures in parentheses are the 3-year average temperature (°C)

### Identification of suitable CMG cultivation sites and comparative evaluation

**Fig. 3:**
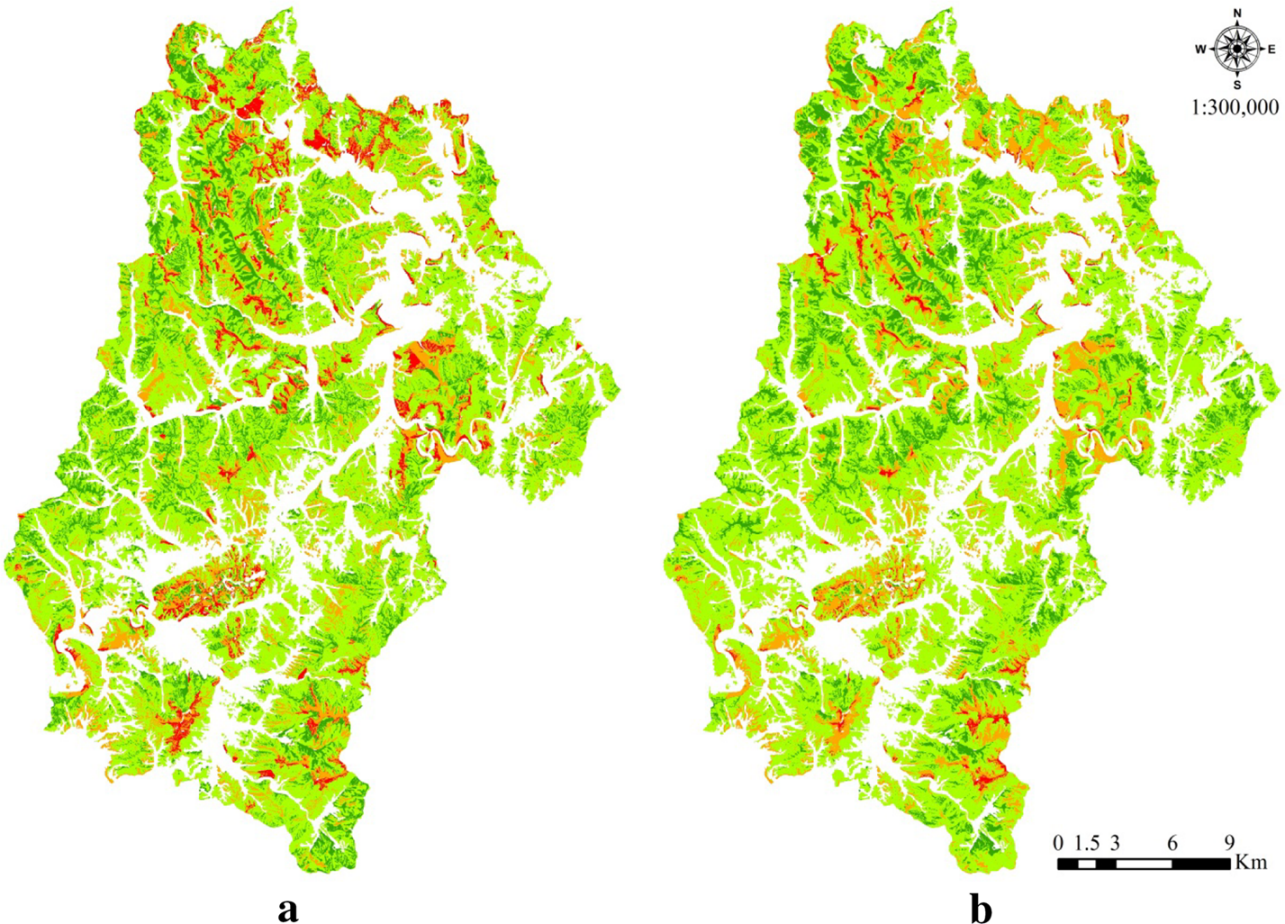
Suitable site maps using the conventional factors (**a**) and the geo-temperature as an additional factor (**b**). *Green*, *yellow*-*green*, *orange*, and *red* indicate suitable site, possibly suitable site, probably unsuitable site, and unsuitable site, respectively

The 2 methods (using the conventional factors, and including the gT as an additional factor) were used to search for suitable sites for CMG cultivation and the results yielded by the 2 methods were compared and evaluated. First, detection using the conventional factors covering the entire forest land in Jinan-gun yielded the following detection performance: suitable, 8209.3 ha (14.1 %); possibly suitable, 36,647.5 ha (65.6 %); probably unsuitable, 8425.15 ha (15.1 %); and unsuitable, 2697.8 ha (4.8 %). Including the gT as an additional factor yielded the following: suitable, 8026.6 ha (14.3 %); possibly suitable, 38,347.1 ha (68.5 %); probably unsuitable, 8587.8 ha (15.3 %); and unsuitable, 1017.9 ha (1.8 %). The area comparison between the 2 methods revealed that including the gT reduced the areas identified as suitable and unsuitable by 0.4 % (182.4 ha) and 3.0 % (1679.9 ha), respectively, and increased the area identified as possibly suitable and probably unsuitable by 3.0 % (1699.6 ha) and 0.2 % (162.7 ha), respectively (Table 3; Fig. 3).

**Table 3:** Site identifications yielded by the 2 methods

Category	Method	
	Application of conventional factors	Addition of geo-temperature
Suitable site	8209.0 (14.7)	8026.6 (14.3)
Possibly suitable site	36,647.5 (65.5)	38,347.1 (68.5)
Probably unsuitable site	8425.1 (15.1)	8587.8 (15.3)
Unsuitable site	2697.8 (4.8)	1017.9 (1.8)
Total	55,979.4 (100.0)	55,979.4 (100.0)

Values are presented as ha (%)

Percentages may not sum to 100 because of rounding

The results using conventional factors were compared with those using gT as an additional factor, by evaluating changes in the search results in each category. The area identified as a suitable site by the conventional factors (66.7 %) was still found to be suitable site when gT was included as an additional factor. However, the remaining area (33.3 %) was demoted into a possibly suitable site when gT was included as an additional factor. The area identified as a possibly suitable site by conventional factors (91.2 %) did not change its classification when gT was included as an additional factor, but changed for the remaining area: 7.0 % as a possibly suitable site, and 1.8 % as an unsuitable site. The area identified as a probably unsuitable site by conventional factors (72.7 %) was still identified as probably unsuitable site when gT was included as an additional factor, but the classification changed for the remaining area: 25.9 % as possibly suitable and 1.4 % as unsuitable. The area identified as unsuitable by conventional factors (33.3 %) was still identified as unsuitable when gT was included as an additional factor, but the remaining area (66.7 %) was identified as probably unsuitable with gT as an additional factor. In summary, 31.8 % of the suitable sites (2550.4 ha), 12.8 % of the possibly suitable sites (4916.7 ha), 28.7 % of the probably unsuitable sites (2466.3 ha), and 11.8 % of the unsuitable sites (119.8 ha) were newly categorized when gT was used as an additional factor. Approximately one-third of the area identified as suitable when gT was used as an additional factor (2550.4 ha) was previously classified as possibly suitable by conventional factors. In addition, approximately one-third of the suitable sites identified by conventional factors (2732.8 ha) was newly classified as possibly suitable. Approximately two-thirds of the unsuitable sites identified by conventional factors (1799.7 ha) were re-categorized as probably unsuitable (Table 4). This illustrated the significant impact of gT as a factor in GIS-based searches for suitable cultivation sites of CMG.

**Table 4:** Cross table for comparison of search results by two different methods

CF^a^	GT^b^				
	Suitable site	Possibly suitable site	Probably unsuitable site	Unsuitable site	Total
Suitable site	5476.2 (66.7)	2732.8 (33.3)	0.0 (0.0)	0.0 (0.0)	8209.0 (100.0)
Possibly suitable site	2550.4 (7.0)	33,430.5 (91.2)	666.7 (1.8)	0.0 (0.0)	36,647.5 (100.0)
Probably unsuitable site	0.0 (0.0)	2183.8 (25.9)	6121.4 (72.7)	119.8 (1.4)	8425.1 (100.0)
Unsuitable site	0.0 (0.0)	0.0 (0.0)	1799.7 (66.7)	898.1 (33.3)	2697.8 (100.0)
Total	8026.6	38,347.1	8587.8	1017.9	55,979.4

Values are presented as ha (%)

Percentages may not sum to 100 because of rounding

^a^CF is the detection result by using conventional factors

^b^gT is the detection result by using geo-temperature as an additional factor

Finally, sites identified as suitable by the 2 methods were extracted and compared with actual CMG cultivation sites (53 sites, 599.0 ha). Using the conventional method, the overlap between identified sites and actual sites was 97.7 ha (16.3 %). When the gT was included as an additional factor, the overlap was 118.9 ha (19.8 %) (Table 5). Although the area identified as suitable by the new method was about 0.4 % (182.4 ha) less than with the conventional method (Table 3), the overlap with actual sites was higher by 3.5 % (21.2 ha) (Table 5). This implies that the new method is more accurate in detecting suitable sites. However, the overlap between the sites identified as suitable and the actual CMG cultivation sites was <20 %. This may be explained by the fact that, owing to the generally small-scale forest land ownership (average 1.8 ha per owner, 56 % of the owners possessing areas under 0.5 ha), CMG cultivation areas owned by forest land owners are accordingly small. The other reason is the expansion of CMG cultivation to inferior growing environments to enhance income. To counteract this trend, the South Korean government began to distribute CMG Cultivation Guidelines in 2013 (Jeon et al. 2013), and has been promoting CMG cultivation revitalization through training and education.

**Table 5:** Overlap ratio between sites identified as suitable and actual CMG cultivation sites

Category	Real cultivation area (a) (ha)	Overlapped area (b) (ha)	Overlap ratio (b/a) (%)
Application of conventional factors	599.0	97.7	16.3
Addition of geo-temperature	599.0	118.9	19.8

## Conclusions

The growth of CMG is influenced by environmental factors such as temperature, humidity, and nutrients. Additionally, further intensive management is required for CMG cultivation as a prominent cash crop compared with timber production (Han 2014). In particular, the first condition for producing high-quality CMG is determining a suitable cultivation site taking into account growth-related and physiological characteristics. In this study, suitable sites were identified by applying the factors used in previous GIS-based studies on the one hand, and by including temperature as an additional influential factor for the different stages of CMG growth. Although the addition of the gT resulted in fewer sites being identified as suitable, the overlap with actual sites was higher, which is indicative of detection accuracy. Moreover, additional sites were identified that did not overlap with the suitable sites identified by the conventional method, which suggests the importance of temperature factors in site detection. Consequently, temperature was found to be important both for detecting suitable CMG cultivation sites and for enhancing the detection accuracy. The main feature of this study is the addition of temperature data, an important factor for CMG growth, to the factors for identifying CMG cultivation sites used in conventional detection methods (Beon et al. 2013; Han 2014). In particular, in countries with high forest-to-land ratio, such as South Korea, the method proposed in this study has great implications for cultivation of CMG and other cash crops by facilitating accurate detection of suitable sites.

In line with the results of this study, to further enhance site detection accuracy, future studies will have to focus on major factors influencing CMG growth among a wide variety of factors related to CMG cultivation. The two methods used for the detection of suitable cultivation sites assigned weighted values on the conditions per each factor. The addition of geo-temperature in the detection of suitable cultivation sites increased the overlap ratio with actual cultivation sites, but the CMG cultivation researchers or cultivators suggest that suitable sites identified by the detection may be possibly suitable or probably unsuitable sites. Similarly, unsuitable sites identified by the detection could be probably unsuitable sites, possibly suitable sites, or even suitable sites. Therefore, underlying certainty as a limitation should be acknowledged despite the scientific and convenient GIS-based method for the detection of suitable cultivation site (Beon et al. 2013). In CMG growth, the relative importance of factors may not be necessarily similar. For example, slope could be twice more important than elevation, or annual average temperature could be have a three times lower importance than temperature in summer. Therefore, future studies could use an analytic hierarchy process (AHP), where the weighted values are assigned to each factor based on the inputs from the experts and researchers of CMG cultivation, to increase the accuracy of the search of suitable cultivation sites, thereby decreasing underlying uncertainty. Accurate identification of suitable CMG cultivation sites is directly associated with enhancing productivity, expanding cultivation areas suitable for normal growth equivalent to the level of suitable sites, and reducing investment loss due to cultivation in unsuitable sites.
